# SiO_2_ nanoparticles modulate the electrical activity of neuroendocrine cells without exerting genomic effects

**DOI:** 10.1038/s41598-018-21157-8

**Published:** 2018-02-09

**Authors:** C. Distasi, F. A. Ruffinatti, M. Dionisi, S. Antoniotti, A. Gilardino, G. Croci, B. Riva, E. Bassino, G. Alberto, E. Castroflorio, D. Incarnato, E. Morandi, G. Martra, S. Oliviero, L. Munaron, D. Lovisolo

**Affiliations:** 1 Department of Pharmaceutical Sciences, University of Piemonte Orientale “A. Avogadro”, Novara, Italy; 2 Department of Life Sciences and Systems Biology, University of Torino, Torino, Italy; 3 Department of Chemistry, University of Torino, Torino, Italy; 4 Center for Synaptic Neuroscience, Istituto Italiano di Tecnologia, Genova, Italy; 5 Human Genetics Foundation, Torino, Italy; 6 NIS Interdepartmental Center, University of Torino, Torino, Italy; 7 Present Address: Department of Neurosciences, University of Torino, Torino, Italy

## Abstract

Engineered silica nanoparticles (NPs) have attracted increasing interest in several applications, and particularly in the field of nanomedicine, thanks to the high biocompatibility of this material. For their optimal and controlled use, the understanding of the mechanisms elicited by their interaction with the biological target is a prerequisite, especially when dealing with cells particularly vulnerable to environmental stimuli like neurons. Here we have combined different electrophysiological approaches (both at the single cell and at the population level) with a genomic screening in order to analyze, in GT1-7 neuroendocrine cells, the impact of SiO_2_ NPs (50 ± 3 nm in diameter) on electrical activity and gene expression, providing a detailed analysis of the impact of a nanoparticle on neuronal excitability. We find that 20 µg mL^−1^ NPs induce depolarization of the membrane potential, with a modulation of the firing of action potentials. Recordings of electrical activity with multielectrode arrays provide further evidence that the NPs evoke a temporary increase in firing frequency, without affecting the functional behavior on a time scale of hours. Finally, NPs incubation up to 24 hours does not induce any change in gene expression.

## Introduction

The fast development of nanoparticles (NPs) designed and engineered to be employed as tools for targeting to specific cells and tissues and for drug delivery has opened an entire new field in both basic science and medical applications. A preliminary, albeit essential, phase was devoted at addressing the concerns about their potential toxicity *in vitro* and, more relevantly, *in vivo*. A huge amount of papers^1–3^ has evidenced the parameters (size, concentration, surface properties, etc.) that can determine the presence or absence of toxic effects, thus providing the rational background for designing safe and biocompatible nanotools. The next step is to switch the focus on the more subtle effects that can arise from a prolonged presence of NPs in contact with cells, and to understand the mechanisms that underlie the interaction between objects at the nanoscale and their cellular and molecular targets. This task is particularly relevant when the target is represented by single nerve cells or by complex neuronal networks.

Engineered silica NPs have encountered a rapid diffusion in several applications in the last decade^4^, specifically in nanomedicine, thanks to the high biocompatibility of this material^5^. We have reported that amorphous 50 nm SiO_2_ NPs can be made fluorescent by hybridization with cyanine dyes and can be safely incorporated into neurons^6^ and other cell types^7^. In a previous paper, we have shown that 50 nm SiO_2_ NPs, at non-toxic doses, elicit increases in the intracellular calcium concentration, [Ca^2+^]_i_, in a neuroendocrine cell line, GT1-7 cells^8^. These signals are fully dependent on calcium influx, carried through different types of calcium permeable channels, and are completely reversible even in the continuous presence of NPs. A few other papers^3,9–11^ have reported that NPs of different composition can elicit changes in neuronal [Ca^2+^]_i_. This is a relevant topic, since perturbations of intracellular calcium homeostasis, even if reversible, can significantly affect neuronal excitability. Electrical activity is the most distinctive functional feature of neuronal cells, and the issue of the potential effects of NPs on the electrical behaviour of neurons and on the ionic channels that underlie it, while still only partially investigated, has attracted increasing interest in recent years, particularly in the light of their value as tools for nanoneuromedicine. Most of these studies deal with the effects of NPs on the properties of a specific channel (see e.g. refs^12,13^). Recently, a few excellent reviews, focused on the potential medical applications of NPs^14–16^, have provided a comprehensive picture of the available knowledge about NPs and neuronal ionic channels; some of them also report the effects on action potential firing. Interestingly, both excitatory^17^ and inhibitory^18,19^ effects have been observed.

A further level of complexity that needs to be addressed stems from the finding that changes in the pattern of neuronal electrical activity may have effects on the expression of relevant genes, particularly when calcium influx is involved^20,21^. In the perspective of using SiO_2_ NPs, both *in vitro* and *in vivo*, for applications such as drug delivery^22^ or cell tracking^23^ in neuronal cells and tissues, it is of the outmost importance to analyze at the cellular level the functional changes induced by the interaction with the NPs, and to investigate whether these changes have stable and long-lasting outcomes on the neuronal phenotype. We have addressed this issue by means of an integrated approach, combining patch-clamp recordings from single neuroendocrine cells and sensory neurons in culture with population recordings by means of multielectrode arrays (MEAs), spanning a time scale from early responses (min) after acute NPs application to hours. In addition, by means of a genomic approach, we have investigated the potential effects of the changes in neuronal activity on gene expression.

## Results

### SiO_2_ NPs interact with the cell membrane and are incorporated in endocytic compartments

**Figure 1:**
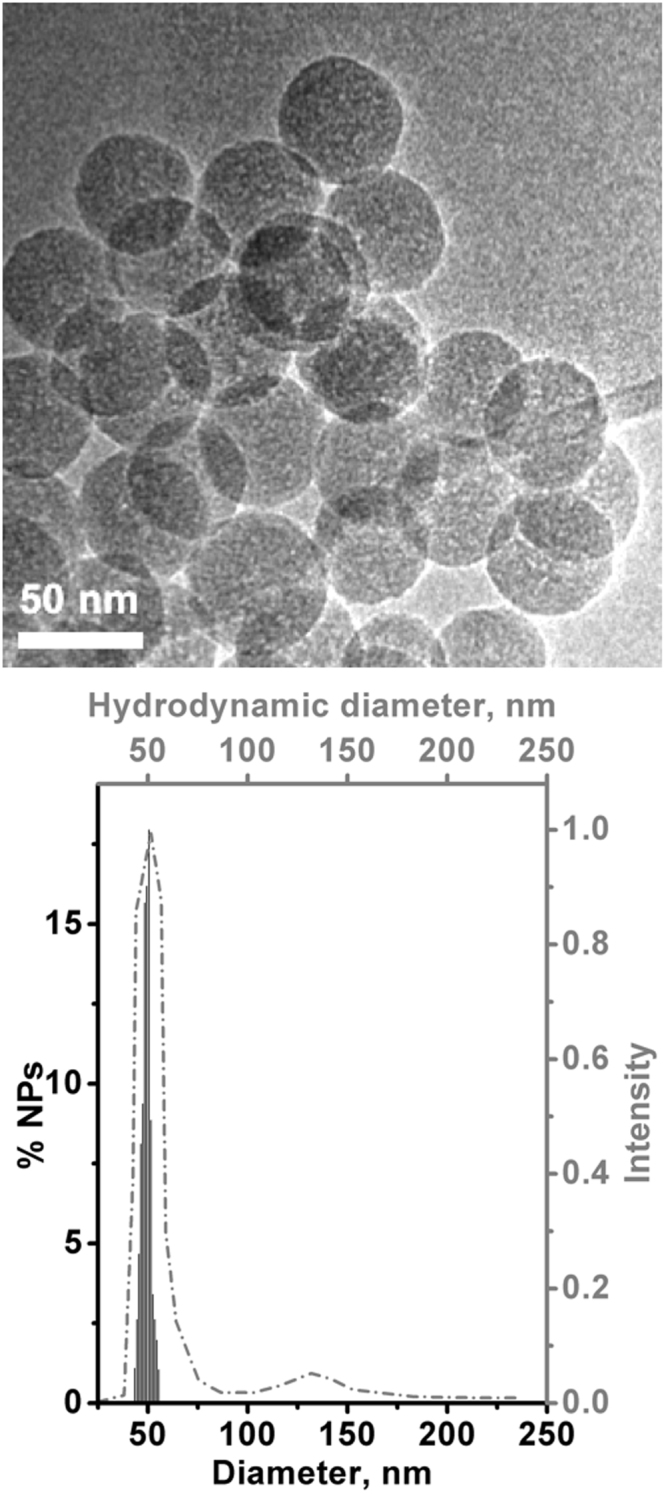
Characterization of the lab-made silica NPs. Upper: representative TEM image (original magnification: X50k). Lower: related particle size distribution by TEM data (histogram; values on the left Y and bottom X axes) and hydrodynamic diameters by DLS in water (dashed-dotted line; values on the right Y and top X axes).

In agreement with previous studies^5,6^ TEM and DLS analysis (Fig. 1) indicated that the lab made SiO_2_ were in the form of nanospheres with a narrow size distribution (mean diameter 50 ± 3 nm). When suspended in water they mainly exhibited a hydrodynamic diameter similar to what observed in TEM images, indicating that they suffered a very limited aggregation.

**Figure 2:**
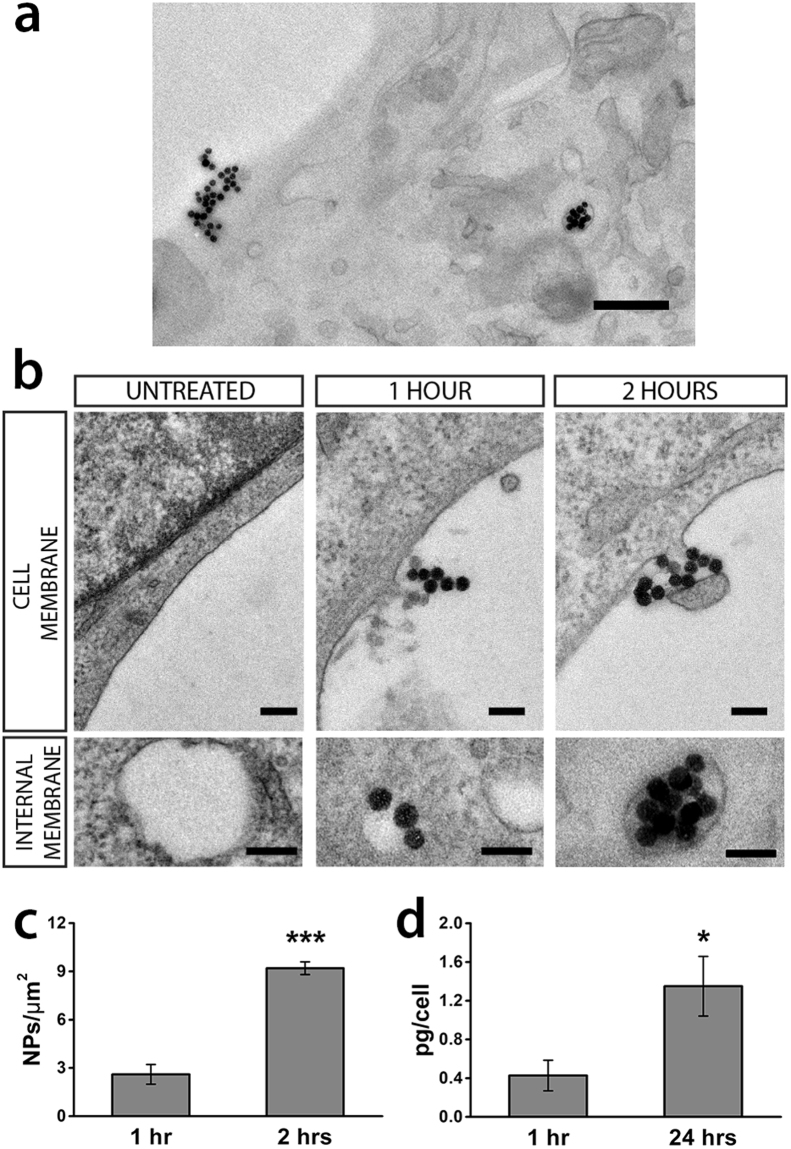
Interaction with the plasmamembrane of GT1-7 cells and internalization of 50 nm SiO_2_ NPs. (**a**) Low magnification TEM image of a GT1-7 cell with NPs on the plasmamembrane and in a vescicle (2 hours incubation). Scale bar: 500 nm. **(b)** After either 1 or 2 hours of incubation, small agglomerates of NPs could be observed in association with the plasmamembrane; some of them were already incorporated into endocytic vesicles. Scale bars: 100 nm. (**c**) NPs density obtained by TEM images at 1 and 2 hrs of incubation. Mann-Whitney U test; ***p < 0.0001 two-tailed, n = 3. **(d)** Quantitative assessment of internalization at 1 and 24 hours of incubation by means of ICP-MS. *t*-test, *p = 0.037 two-tailed, n = 4.

In order to analyze the distribution of nanoparticles on the cell membrane, we imaged by electron microscopy GT1-7 cells exposed for 1 or 2 hours to 20 µg mL^−1^ NPs. Ultrastructural analysis (Fig. 2a,b) reveals that the NPs were present on the plasma membrane (NP density for 1 hour treated cells: 2.60 ± 0.61 NPs µm^−2^; 2 hours treated cells: 9.20 ± 0.39 NPs µm^−2^; Fig. 2c) suggesting that they can interact with membrane channels at times shorter than 1 hour. Following the interaction with the plasma membrane, nanoparticles could be internalized inside the cell. Intriguingly, free nanoparticles were never imaged either in the cytoplasm of the GT1-7 cells or inside mitochondria, Golgi apparatus, endoplasmic reticulum or nucleus, but always inside membranous endocytic-like structures, indicating a possible induction of endocytosis after the interaction with the plasma membrane. A quantitative assessment of NPs incorporation in GT1-7 cells was performed by means of the ICP-MS technique (Fig. 2d). It can be seen that at 1 hour of incubation, a detectable amount of NPs could be measured (0.43 ± 0.16 pg/cell), that was significantly increased at 24 hours (1.35 ± 0.31 pg/cell).

### SiO_2_ NPs elicit depolarizing responses in GT1-7 cells and adult mouse sensory neurons

**Figure 3:**
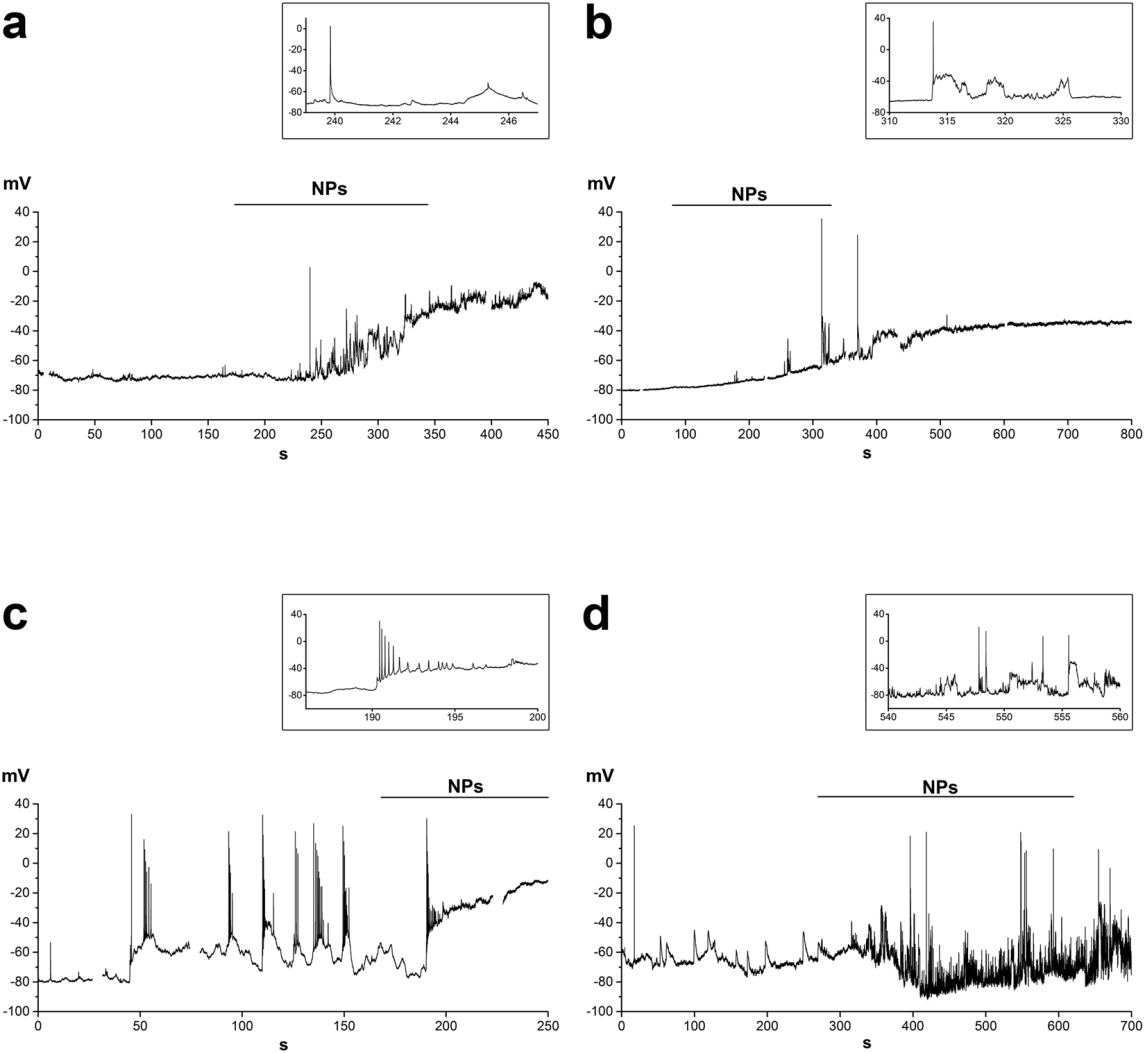
Four examples of the changes in electrical activity recorded from single cells in response to 20 µg mL^−1^ SiO_2_ NPs. Insets show expanded segments at the times indicated. Traces were blanked in correspondence to the application of the voltage clamp protocols as shown in Supplementary Fig. S1.

When bathed in physiological Tyrode solution, GT1-7 cells showed different patterns of electrical activity as recorded in patch clamp experiments, in current clamp mode. Out of 32 cells, 19 had a stable resting potential (Fig. 3a,b), 10 cells showed small oscillations (Fig. 3d); in three cells firing of full-sized action potential was detected (Fig. 3c). Average V_m_ was −69.7 ± 2.2 mV.

The 50 nm SiO_2_ NPs were used at the concentration of 20 µg mL^−1^, that in previous experiments had been shown to be non-toxic^6^ and to elicit strong and long-lasting but reversible [Ca^2+^]_i_ oscillations^7^. When NPs were added to the extracellular Tyrode solution, they elicited a depolarizing response (average ∆V = 40.1 ± 3.1 mV) in 28 out of 32 cells. In most cells, after transient firing of one or few full action potentials, the membrane potential either showed oscillatory activity with small spikes (Fig. 3a) or was stabilized at the depolarized value (Fig. 3b,c), without showing further oscillations and/or spikes, probably because of a strong inactivation of voltage dependent Na^+^ and Ca^2+^ channels. In two cells firing activity was markedly increased for longer times (Fig. 3d).

The depolarizing response was in general irreversible, at least for the duration of the recordings (up to 20 min). The functional state of the cells, checked by transiently switching to the voltage clamp mode and recording responses to voltage stimulation protocols appeared to be preserved (an example, related to the response of Fig. 3b, is shown in Supplementary Fig. S1).

In two experiments, cells were first challenged with a lower dose (0.5 µg mL^−1^) and subsequently with 20 µg mL^−1^. Supplementary Fig. S2 shows that the lower dose induced a small and reversible depolarization, while the response to the higher one was stronger and oscillatory.

**Figure 4:**
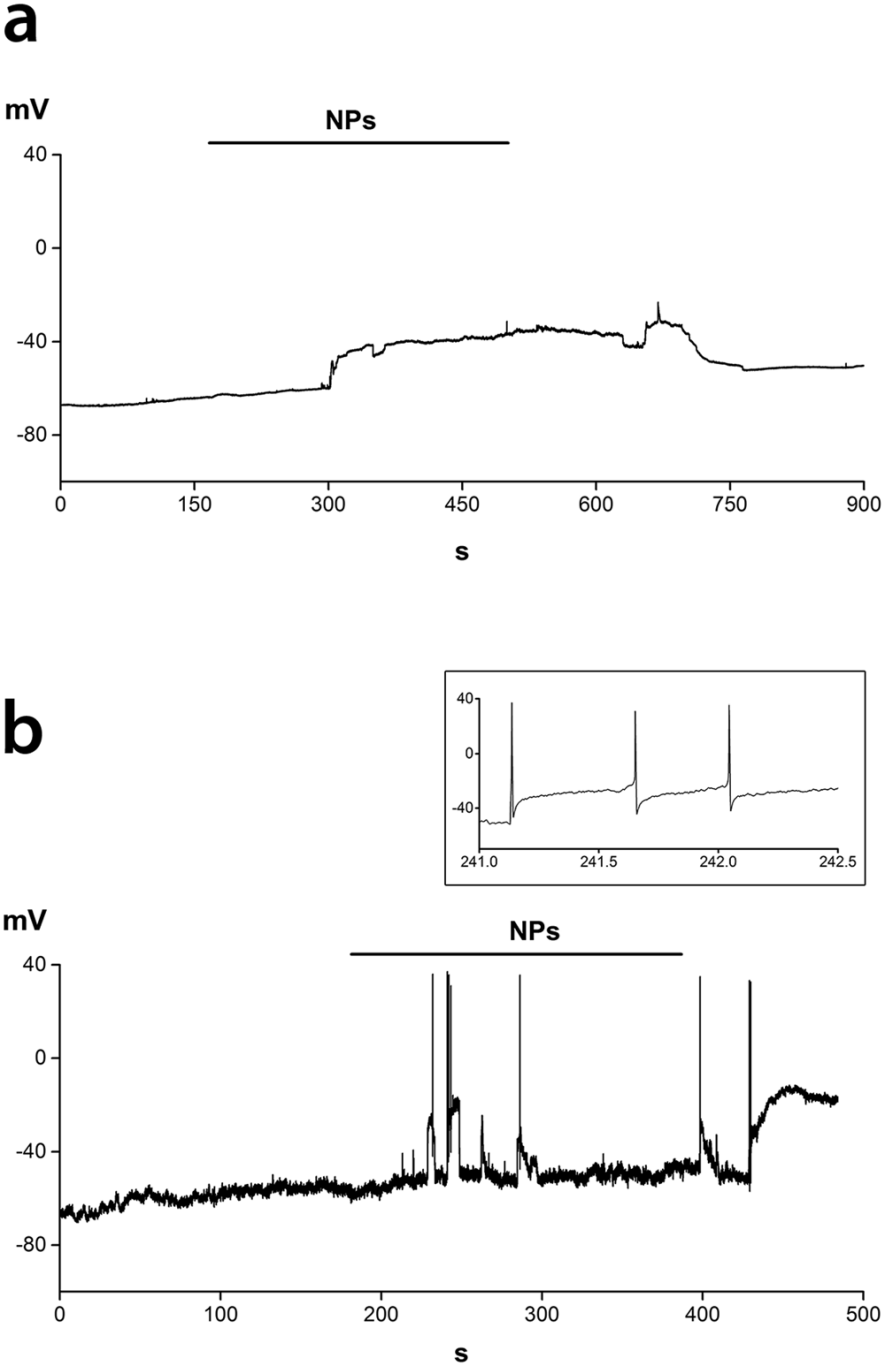
Current clamp recordings from mouse DRG neurons. (**a**,**b**) Two examples of changes in V_m_ induced by 20 µg mL^−1^ SiO_2_ NPs. Inset in (**b**) shows action potential firing at expanded time scale.

These findings were confirmed by recordings from another neuronal model, primary cultures of dorsal root ganglion (DRG) sensory neurons from adult mice. DRG neurons had a stable resting potential of −60.7 ± 1.5 mV (n = 20), ranging from −72.0 mV to −47.0 mV. Only 30% of neurons generated sporadic spontaneous activity. In all cells challenged with 20 µg mL^−1^ 50 nm SiO_2_ NPs (n = 8), we recorded a depolarizing response (average ∆V = 37.8 ± 3.0 mV, ranging from 27.5 to 53.8 mV). In most cells, the membrane potential shifted to a depolarized value without firing any action potential, as shown in Fig. 4a. The remaining two neurons, prior to the V_m_ stabilization at the depolarized value, fired one or a few action potentials in response to fast and transient V_m_ changes above threshold (Fig. 4b).

### SiO_2_ NPs transiently increase the electrical activity of GT1-7 cells in long lasting recordings

To provide further information on the electrical activity elicited by the NPs on a longer time scale, comparable to the duration (several hours) of the calcium imaging experiments reported in a previous paper^7^ and at a population level with a non-invasive approach, we performed extracellular recordings from GT1-7 cells cultured on MEAs.

**Figure 5:**
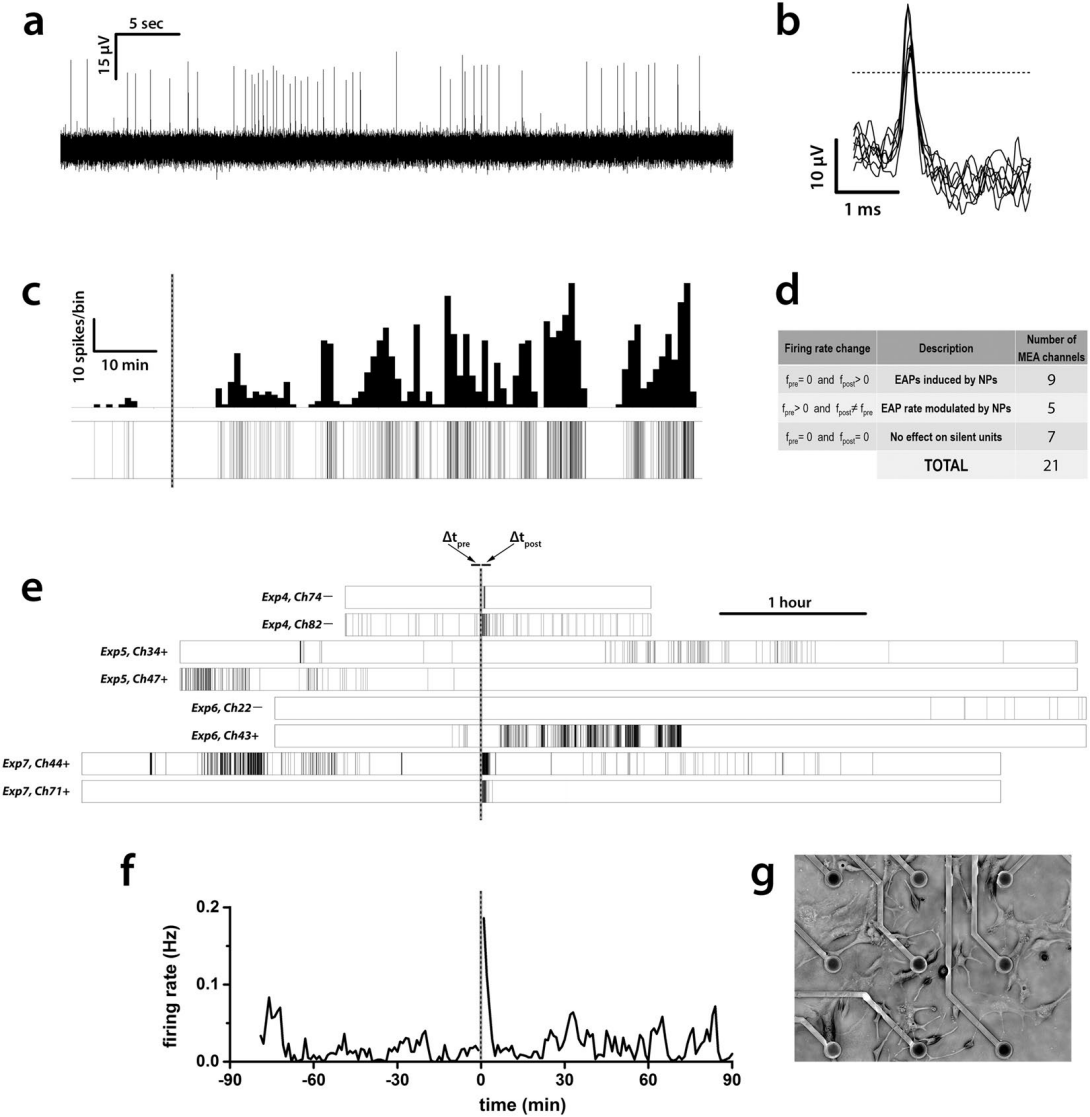
MEA recordings. (**a**) A representative raw signal featuring many EAPs, as displayed by the acquisition software MC_Rack (sampling frequency: 25 kHz). (**b**) 7 superimposed waveforms consisting of upward EAPs extracted from raw data. Each cutout extends from 1 ms before to 2 ms after the point where the signal crosses the threshold (horizontal dashed line) with a negative slope. Threshold was fixed at ± 6 standard deviations from the baseline noise. (**c**) A representative raster plot (lower trace) and the corresponding rate histogram (upper trace) of an active channel (the same shown in (**e**): Exp6, Ch43+). Bin size: 60 s. 10 spikes/bin ≈ 0.17 Hz. Dashed line is the time of NPs administration. (**d**) Schematic overview of the early changes induced by 20 µg mL^−1^ SiO_2_ NPs in GT1-7 EAP rate. Table refers only to the short temporal window (Δt_pre_ + Δt_post_) as shown in (**e**). (**e**) 8 complete raster plots (representative of 21) from the 7 experiments featuring the active channels used for the analysis. On the left side of each raster are the experiment number and the channel reference. Symbols + and − represent the direction (upward or downward respectively) of the EAPs detected. Dashed line is the time of medium change. (**f**) Global rate histogram resulting from the average of the rate histograms of all the 21 active units. Here, too, the dashed line is the time of medium change and NPs administration. **(g)** Low magnification image of GT1-7 cells pated on MEAs.

Full recordings sampled at 25 kHz (Fig. 5a) were filtered using a voltage threshold (see Methods) in order to extract extracellular action potential (EAP) waveforms from raw data (Fig. 5b), and build raster plots (Fig. 5c, lower) and rate histograms (Fig. 5c, upper). Rate histograms represent the time course of the firing rate and they can be used to highlight how the electrical activity clusters in time. In our experiments, both under control condition and in presence of NPs, GT1-7 cells exhibited a characteristic firing pattern, in which active and resting phases alternated with an irregular period ranging from 5 to 15 min (Fig. 5c). This is in good agreement with the reported behavior of GT1-7 cells described in literature^24^.

Over 24 different MEAs, just 7 (about 30% of the total) showed a sustained electrical activity in at least one MEA electrode. For each of these experiments, an average of 3 out of 59 electrodes (5%) recorded non-sporadic activity, resulting in a total of 21 independent long duration recordings. Half of them were silent channels under control conditions (standard physiological solution) that started to show electrical activity only after 20 µg mL^−1^ NPs administration (Fig. 5e, *e.g*. Exp7, Ch71+). Conversely, the other half presented some degree of spontaneous activity already under control conditions and the effect of NPs on these channels apparently consisted in a modulation of the number of EAPs recorded (Fig. 5e, *e.g*. Exp4, Ch82–), confirming the heterogeneity already observed in current clamp experiments.

In order to quantify the overall effects of NPs on GT1-7 firing rate, distinguishing between early and long lasting effects, we firstly decided to measure the average number of EAPs within two temporal windows of equal length (200 s), placed just before (Δt_pre_) and immediately after (Δt_post_, Fig. 5e) NPs administration. Within these temporal borders, active channels changed their firing rate according to Fig. 5d.

Overall, the average firing rate under control conditions (average spontaneous activity) was <f_pre_> = 0.014 ± 0.011 Hz, that increased to <f_post_> = 0.095 ± 0.038 Hz after NPs addition. The maximum value for GT1-7 firing rate we recorded (using a 1 min binning) was f_max_ = 1.02 Hz, but both f_max_ and <f_post_> are possibly underestimated because of the blind spot (about 15 seconds) in MEA recording due to medium substitution. To compare the differences in firing rate in the two conditions, a paired-samples sign test was used. The sign test is a non-parametric alternative to the paired-samples *t*-test and Wilcoxon signed-rank test, being our distribution of differences Δf = f_post_ − f_pre_ neither normal nor symmetrical. NPs elicited a statistically significant median increase in firing rate compared to the spontaneous electrical activity (*p* = 4.883·10^−4^ two-tailed, n = 21). Different choices for window length (*e.g*. Δt = 400 s, Δt = 500 s) led to the same conclusions, even though *p*-values showed a clear increasing trend as window length increased (*p* = 0.002 for Δt = 400 s; *p* = 0.013 for Δt = 500 s), thus confirming the transient nature of the effect on the firing rate elicited by NPs. In particular, after about 10 min from NPs administration, this effect on GT1-7 firing rate was no more detectable in any of the channels taken into account, even if nearly half of them still showed some kind of electrical activity after 1 hour or more. The late firing rate of this portion of channels, such as their percentage respect to the total of the active channels used for the analysis, were comparable to the ones measured under control condition. A global firing rate histogram (Fig. 5f) was drawn by averaging the full rate histograms from all the 21 active channels. It shows that, after the short and early phase already discussed, electrical activity indeed recovered to control levels and was preserved in time, thus providing evidence that 50 nm SiO_2_ NPs, at the concentration of 20 µg mL^−1^, do not exert long lasting effects on neuronal function. Figure 5g shows an image of the GT1-7 cells plated onto the MEAs.

### Incubation with NPs up to 24 hours does not affect gene expression

**Figure 6:**
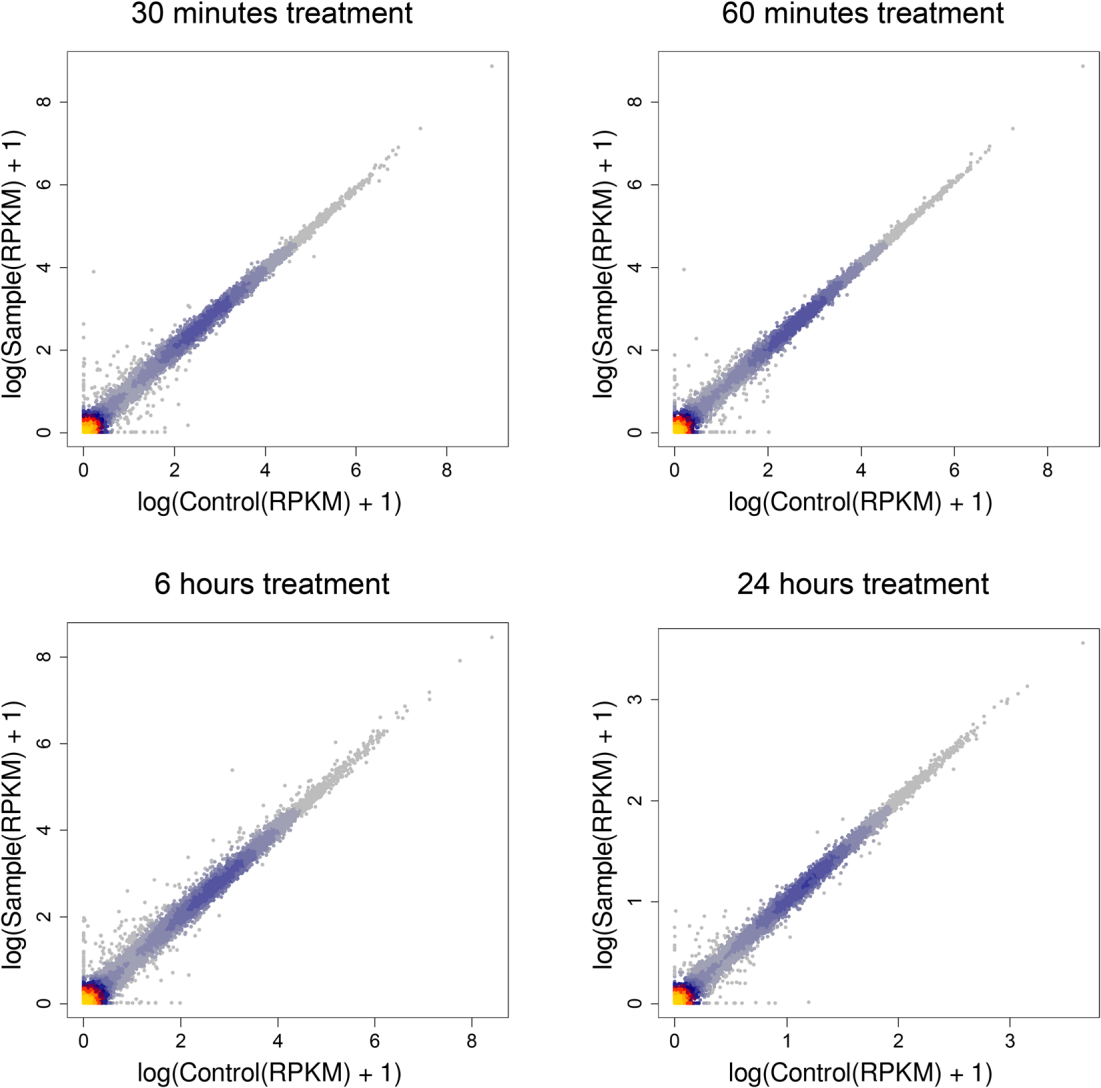
Lack of detectable transcriptional responses to incubation with SiO_2_ NPs. Scatter plot with heat density of pairwise expression level comparison between treated and untreated (20 µg mL^−1^ of 50 nm SiO_2_ NPs) cells at 30 minutes, 1, 6 and 24 hrs post-treatment. Gene expression values (per kb per million total reads, RPKM) in log scale. Dots indicate each gene expressed in the two samples.

To further characterize the effect of SiO_2_ NPs treatment, and evaluate whether it could induce a transcriptional response, we performed RNA-Seq in GT1-7 cells at various time points following treatment with 20 µg mL^−1^ NPs. RNA-Seq analysis of the samples at 30 min, 1, 6 and 24 hrs showed very high correlation (*R* > 0.99, Pearson Correlation Coefficient) between treated and untreated cells for the entire timescale of the analysis (Fig. 6), revealing that the SiO_2_ NPs do not induce a detectable transcriptional response.

Since these experiments were performed in the absence of FBS, and since the lack of toxicity of the concentration of 20 µg mL^−1^ has been tested in a previous paper^6^ in the presence of 0.5% FBS, we repeated the survival assay at 24 hours on cells cultured in DMEM without FBS. Supplementary Fig. S3 shows that also under these conditions the NPs did not affect cell survival.

## Discussion

The data presented in this paper show that our nanoparticle model, 50 nm SiO_2_ NPs, non-toxic at the size and concentration employed, induces fast changes in membrane potential and modulates the electrical activity of GT1-7 cells. MEAs recordings provide evidence for a transient increase in the firing rate of action potentials, without inducing long lasting alterations on the time scale of a few hrs. This is in agreement with previous observations^8^ that the increases in [Ca^2+^]_i_ induced by the presence of these NPs were transient and reversible. These findings are not limited to a specific experimental model. We have observed similar responses to 50 nm SiO_2_ NPs with single cell recordings from primary adult neurons that have electrical properties quite different from GT1-7 cells: the latter, in varying percentages according to culture conditions, are able to generate spontaneous electrical activity, either sporadic or in bursts^25^; DRG neurons, on the other hand, even if representing an heterogeneous population of different sensory cells, have a very limited and sporadic spontaneous electrical activity^26^.

Interestingly, ultrastructural and ICP-MS data provide evidence for an interaction with the plasma membrane followed by the activation of an endocytic process. It is of relevance that in the culture medium, NPs form very small agglomerates, as reported by dynamic light scattering (DLS) data in a previous paper^8^.

A very recent paper^27^, combining a complex set of experimental approaches with numerical simulations, has reported that different kinds of nanoobjects (Quantum Dots, Quantum Rods, Fe_2_O_3_ NPs of different shape) elicit an increase in electrical activity in cultured rat hippocampal neurons. The most relevant and novel findings are: i) the responses are size-and shape independent; ii) electrical charge is the crucial parameter: negative NPs adhere to the neuronal plasmamembrane, cause a depolarization and increase firing, neutral and positive ones do not; iii) NPs interact only with active neurons. While in the present paper we did not characterize the dependency of the responses from these parameters, in a previous paper we have shown that SiO_2_ NPs with a negative ζ potential elicited increases in [Ca^2+^]_i_, while positively charged (aminated) ones had no effect. In the above paper, we reported a size dependency; however the size range (50–200 nm) was broader than the range (5–75 nm) tested in ref.^25^. While the issue of dependency of interaction with the plasmamembrane from the negative surface charge of NPs finds support in the data from several other laboratories (see e.g. refs^28,29^), the independency from size needs further investigation. Finally our experiments show that, in both GT1–7 and DRG neurons, the firing activity is not a requisite for SiO_2_ NPs-neuronal membrane interaction.

The fact that electrical activity in neurons may have effects on gene transcription (the “excitation-transcription” paradigm^14,15^), mainly because of its link to changes in [Ca^2+^]_i_, were the rational for carrying out the analysis of the whole transcriptome. Interestingly, we found that the treatment with SiO_2_ NPs does not induce a detectable transcriptional response at all time points analyzed, and this deserves further comments.

The issue of the effects of NPs on the target cell genome has attracted increasing interest in the last decade, the main focus being on potential genotoxic effects. Several comprehensive reviews are available^30–32^; in general, evidence for DNA damage induced by SiO_2_ NPs is limited, and confined to relatively high doses (in most cases over 100 µg mL^−1^). Most of the reported effects are cell- and nanoparticle size specific (the smaller being in general more genotoxic), thus requiring great caution when trying to derive general conclusions. Data based on neuronal models are more scarce, and limited to the pheochromocytoma cell line, PC12 cells. Here the reports are conflicting: it has been found^33^ that 20 nm SiO_2_ NPs at the concentration of 500 µg mL^−1^ did not alter chemokine and NF-ĸB levels in microglial cell, but only induce a small increase in the secretion of inflammatory chemokines; the supernatant from these cells, when applied to PC12 cells, did not induce cytotoxicity nor changes in a few marker genes. On the other hand, with 50 µg mL^−1^ of 25 nm NPs, other Authors^34^ found activation of reactive oxygen species (ROS) and aggregation of α–synuclein, a potential early Parkinsons’ Disease marker. Finally another group^35^, using 20 and 50 nm NPs, report that they affect viability in a time dependent way at concentrations starting from 50 µg mL^−1^ for the former and from 100 µg mL^−1^ for the latter; both induced significant increases of ROS starting from 25 µg mL^−1^. A likely explanation for these discrepancies can be based on the different sizes employed, and, more relevantly, from the different experimental protocols: in the first two cases the NPs were administered to PC12 cells in the presence of 10% FBS, while in the latter stimulation was performed on cells in 0.1% FCS. This comparison points to another key issue in analyzing toxicity and genotoxicity induced by NPs: it has been reported that the toxic doses can change even of a few orders of magnitude when evaluated in the presence or in the absence of serum^36^. The finding that in our experimental conditions, the lack of genomic effects is paralleled by a lack in reduction of cell survival, even in the absence of FBS, strengthens the conclusion that the use of these particles is particularly safe and opens to the possibility of employing them as markers for cell tracking and/or carriers for drug delivery. This is, to our knowledge, the first detailed description of the changes in electrical activity of neurons following exposure to a nanoparticle, and of its evolution in time. The transient nature of these effects can explain the absence of long term outcomes on cell fate.

## Materials and Methods

### Materials

Unless otherwise specified, materials were obtained from Sigma Aldrich.

### Synthesis of silica NPs

Synthesis and characterization of the SiO_2_ nanoparticles have been described in previous papers^6,8^. Briefly, bare silica nanoparticles were synthesized by the reverse microemulsion technique; in particular, a reverse microemulsion was prepared by mixing 75.0 mL of cyclohexane, 18.85 g of Triton X-100, 18.0 mL of n-hexanol and 5.4 mL of deionized H_2_O in a round bottom flask. After 30 min of equilibration at room temperature and continuous gentle magnetic stirring, 1.0 mL of tetraethylorthosilicate (TEOS) was added; after further 10 min of equilibration, reaction was started by the addition of 0.7 mL of NH_3_ (28–30%) and continued overnight at room temperature under magnetic stirring. After 16 hrs, reaction was interrupted by the addition of acetone; then particles were extracted by centrifugation at 10,000 rpm for 20 min and finally washed twice with ethanol and several times with deionized water until complete removal of surfactant. The nanoparticles were stored in water suspension (1 mg mL^−1^) at room temperature.

### High resolution transmission electron microscopy (HRTEM)

Micrographs of nanoparticles were acquired using a 3010 Jeol instrument operated at 300 kV. Samples were prepared by spreading a droplet of the corresponding suspensions in MilliQ H_2_O (1.0 mg mL^−1^) on a copper grid coated with a lacey carbon film and then letting water to slowly evaporate to limit particle agglomeration. The mean particle diameter was calculated as d_m_ = Σd_i_n_i_/Σn_i_ (n_i_ = number of particles of diameter d_i_) by measuring the size of ca. 300 particles.

### Dynamic Light Scattering (DLS)

DLS measurements were performed in a 90Plus Particle Size Analyzer (Brookhaven Instruments) at a laser wavelength of 660 nm, a detection angle of 90°, and 293 K. Samples were prepared by suspending NPs in Milli-Q water (pH 5.5, concentration: 0.1 mg mL^−1^). DLS plots are reported in number distribution.

### GT1-7 cell cultures

GT1-7 cells, an immortalized line derived from highly differentiated mouse gonadotropin-hormone releasing hormone (GnRH) neurons (generously donated by Prof. P.L. Mellon), were maintained in Dulbecco’s Modified Eagle’s medium (DMEM) supplemented with 10% fetal bovine serum (FBS, Lonza), gentamycin (50 µg mL^−1^), and glutamine (2 mM) at 310 K, in a humidified atmosphere of 5% CO_2_ in air. For patch-clamp experiments, cells were plated at the density of 9,000 cells cm^−2^ on uncoated plastic dishes (Corning). After 24 hrs, medium was changed to 0.5% FBS supplemented with 1/50 of B27 (Invitrogen) to improve survival and differentiation, then switched to 0.5% FBS alone for 4–6 days for patch-clamp experiments (a protocol similar to that described in ref.^25^) or 8–15 days for multielectrode arrays (MEAs) experiments. Plating density was increased to 20,000–30,000 cells/cm^2^ both for genomic experiments, in order to have a sufficient amount of cells for RNA extraction, and for recordings on MEAs, to reach a higher surface of microelectrodes covered by cells.

### Isolation and Primary Cell Culture of Mouse Dorsal Root Ganglion Neurons

Mice were purchased from Charles River (Segrate, Italy). The care and husbandry of mice were in conformity with the institutional guidelines in compliance with national (D. L.vo 26/2014, Gazzetta Ufficiale della Repubblica Italiana, n.61, March 14th 2014) and international laws and policies (European Union directive 2010/63/UE; Guide for the Care and Use of Laboratory Animals, U.S. National Research Council, 1996). Adult Balb-C mice (5/10-wk-old) were euthanized under deep isoflurane-induced anaesthesia. The procedures were approved by the local animal-health and ethical committee (Università del Piemonte Orientale) and were authorized by the national authority (Istituto Superiore di Sanità; authorization number N. 22/2013).

DRG were aseptically removed and collected in a dish containing cold F12 (Nutrient Mixture F12 Ham) medium. Working under a dissecting microscope and using fine forceps, the surrounding membrane was gently teased away from each DRG; nerves and sheath were cut. All desheathed DRG were then transferred into a sterile 35 mm dish containing collagenase from clostridium hystoliticum 0.125% and DNase in F12 medium and incubated at 310 K for 1 hour. After incubation, DRG were triturated using a tip p1000. Myelin and nerve debris were eliminated by centrifugation trough a bovine serum albumin (BSA) cushion. Cell pellet was re-suspended in Bottenstein and Sato medium (BS) (30% F12, 40% DMEM (Dulbecco’s Modified Eagle’s medium, 30% Neurobasal A medium (Life Technologies, Italy), 100 x N2 supplement (Life Technologies, Italy), penicillin 10 U/mL and streptomycin 100 mg/mL, supplemented with Recombinant Human β-NGF, Recombinant Murine GDNF and Recombinant Human NT3 (Peprotech, USA) and plated onto 35 mm Petri dish pre-coated with laminin. Two days after, electrophysiological patch clamp measurements were performed on DRG neurons of all soma sizes.

### Exposure to NPs

Prior to the experiments, cells were switched to the appropriate extracellular medium (either DMEM or standard physiological solution, both serum-free). NPs, stored in a stock solution (1 mg mL^−1^ in water) were sonicated for 20 min before final dilution. For all protocols except patch-clamp, the NPs were administered by substituting the extracellular solution with an identical one containing a stable suspension of NPs at the final desired concentration.

### Incorporation of nanoparticles

For conventional transmission electron microscopy (TEM), GT1-7 cells were treated for 1 and 2 hours with NPs and then fixed with 1.2% glutaraldehyde in 66 mM sodium cacodylate buffer, post-fixed in 1% OsO_4_, 1.5% K_4_Fe(CN)_6_, 0.1 M sodium cacodylate, *en bloc* stained with 1% uranyl acetate, dehydrated, and flat embedded in epoxy resin (Epon 812, TAAB). After baking for 48 hrs, the glass coverslip was removed from the Epon block by thermal shock. Cells were identified by means of a stereomicroscope, excised from the block and mounted on a cured Epon block for sectioning using an EM UC6 ultramicrotome (Leica Microsystem, Vienna). Ultrathin sections (70–90 nm thick) were collected on copper mesh grids and observed with a JEM-1011 microscope (Jeol, Tokyo, Japan) operating at 100 kV and equipped with an ORIUS SC1000 CCD camera (Gatan, Pleasanton, CA). For each experimental condition, at least 100 images were acquired at 10,000 to 15,000x magnification and analyzed using the ImageJ software^37^.

Si^2+^ uptake was quantified by using inductively coupled plasma mass spectrometry (ICP-MS; element-2; Thermo-Finnigan, Rodano (MI), Italy). GT1-7 cells were seeded at the initial density of 20.000 cells cm^−2^ in DMEM 10% FCS. The following day, the medium was changed with DMEM 0.5% FCS supplemented with B27 to improve survival and differentiation. Afterward, cells were incubated with SiO_2_ NPs (20 µg mL^−1^) added to DMEM 0.5% FCS for 1 and 24 hrs. After the treatments cells were washed for three times with PBS solution, detached with Trypsin, recounted and collected into an appropriate conical tube and centrifuged (5 minutes at 1000 rpm). The supernatant was discarded and the pellet was analyzed with ICP-MS. Sample digestion was performed with concentrated HNO_3_ (70%, 1 mL) under microwave heating at 433 K for 20 minutes (Milestone MicroSYNTH Microwave labstation). Control measurements were performed as above but omitting nanoparticles; values were below the sensitivity of the instrument.

### Electrophysiology - Patch-clamp

Conventional whole cell patch-clamp recordings were performed in the current clamp mode at 295–298 K. Cells were continuously superfused with a standard physiological solution of the following composition (in mM): NaCl, 154; KCl, 4; CaCl_2_, 2; MgCl_2_, 1; 4-(2-hydroxyethyl)-1-piperazine ethane sulfonic acid (HEPES), 5; glucose, 5.5; and NaOH (pH 7.35). Composition of the pipette solution was (in mM): KCl, 15; CaCl_2_, 3; MgCl_2_, 3; Hepes, 5; KAsp, 118; EGTA, 5; Na_2_ATP, 5; pH to 7.3 with KOH. Pipettes had a resistance of 2–5 MΩ. NPs were dispersed in the solutions at the required concentration. Solutions were applied by means of a microperfusion system connected to a set of five syringes containing the control and test solutions; the perfusion pipette was located at several tens of microns away from the cell to be recorded, in order to minimize mechanical perturbations. Correction for junction potential was performed analogically. Data were collected with an Axopatch 200B amplifier (Molecular Devices, USA) using Clampex 10.2 and Axoscope 10.2 software. Step protocols, in the voltage clamp mode, were applied to check cell functionality. In the presence of NPs, whole cell recordings lasted from 5 to 30 min, durations comparable to those obtained in control experiments in the absence of NPs.

### Electrophysiology - Multielectrode Arrays (MEAs)

Extracellular GT1-7 action potentials were recorded by means of a commercial 60-channel multi-electrode array (MEA) setup. A description of the experimental apparatus is provided in Supplementary Methods online. An external T-control unit (TC02) kept the temperature of the socket surface at 310 K. The digitalized output-data coming from the USB-ME64 unit were acquired, monitored and recorded on a PC through MC_Rack software (Version 4.6.2, Multi Channel Systems MCS GmbH). To reduce the data file size, only upward (positive) and downward (negative) spike cutouts from each of the available recording electrodes were stored. These waveforms consisted of 1 ms pre-spike and 2 ms post-spike fragments respect to the point of threshold crossing. Threshold was fixed at ± 6 standard deviations from baseline noise (typically around 15 µV peak-to-peak). A channel was considered active if it recorded at least 30 spikes in 5 hrs. In general, according to the technical specifications of the device, one electrode does not correspond to a single cell. Rather, each electrode can detect all the electrical signals originating in the spatial region surrounding it, within a radius of tens of micrometers. Waveforms drawn from each active channel were finally transformed into time stamps using NeuroExplorer (Nex Technologies, USA). The same software was used also to make raster plots and rate histograms.

### Survival assay

Cytotoxicity was evaluated by counting cells with a Burker chamber at 24 hours after incubation in DMEM medium supplemented with B27 without FBS either in the presence or in the absence of the NPs. Data are expressed as number of cells/cm^2^.

### RNA-Seq analysis

GT1-7 cells, plated in 10 cm Petri dishes and kept in 0.5% FBS for 4 days, were shifted to DMEM without FBS to prevent adsorption of serum proteins to NPs and either treated or not (control cells) with 20 µg L^−1^ NPs for 30 min, 1, 6 and 24 hrs. Cells were then washed with ice cold PBS and lysed with TriReagent (Sigma), 1 mL per dish, harvested with a scraper and stored at 193 K until RNA extraction. RNA was purified as previously described^38^. RNA integrity measurements were performed using Fragment Analyzer™ (Advanced Analytical). All samples had RNA Quality Number (RQN) greater than 9.8. RT-qPCR was performed using the SuperScript III Platinum One-Step Quantitative RT-PCR System (Invitrogen) following the manufacturer’s instructions. Library preparation was performed using Illumina TruSeq RNA prep-kit following manufacturer’s instructions. Reads were mapped using TopHat2^39^ and RPKM were calculated using cuffdiff tool from Cufflinks software package^40^. Data analysis and plotting was performed using custom R scripts^41^. RNA-seq data has been deposited on Gene Expression Omnibus database under the accession GSE98032.

### Statistical analysis

Particle size evaluated by TEM is given as mean ± SD. Unless otherwise specified, all other data are expressed as means ± SEM. For analysis of NP-cell internalization, differences were assessed by a Mann-Whitney U test; for ICP-MS measurements and cell survival assays, differences were checked by a *t*-test (after normality had been verified through a Shapiro-Wilk test); in the analysis of MEAs recordings, to compare the differences in firing rate, a paired-samples sign test was used.

### Data availability

The RNA-seq data has been deposited on Gene Expression Omnibus database under the accession GSE98032. All other datasets generated and analysed during the current study are available from the corresponding author on reasonable request.

## Electronic supplementary material

Distasi et al. - Supplementary Information
