# Supplementary material for: SiO2 nanoparticles modulate the electrical activity of neuroendocrine cells without exerting genomic effects
Source: Sci Rep. 2018 Feb 9;8:2760. doi: 10.1038/s41598-018-21157-8 (PMC5807366; doi:10.1038/s41598-018-21157-8)
Supplement: Supplementary file 1 — Distasi et al. - Supplementary Information [file 41598_2018_21157_MOESM1_ESM.pdf]

Carla Distasi, Federico A. Ruffinatti, Marianna Dionisi, Susanna Antoniotti, Alessandra Gilardino, Giulia Croci, Beatrice Riva, Eleonora Bassino, Gabriele Alberto, Enrico Castroflorio, Danny Incarnato, Edoardo Morandi, Gianmario Martra, Salvatore Oliviero, Luca Munaron, Davide Lovisolo

SiO_2 nanoparticles modulate the electrical activity of neuroendocrine cells without exerting genomic effects

Supplementary Information

Methods

MEA recordings

The USB-MEA60-Inv-BC-System by Multi Channel Systems MCS GmbH consisted of a probe interface with an integrated 60-channel pre-amplifier (MEA1060-Inv-BC-PA; Gain: 1100) and a bandpass filter amplifier (FA60S-BC; Bandwidth: 1Hz-3kHz). This stage was connected to an analog-to-digital converter board, specifically designed for data acquisition and computer interfacing (USB-ME64; 25kHz sampling rate per channel and 16-bit resolution). Each probe was a 60MEA200/30iR-Ti-gr, namely a squared glass plate containing 60 planar micro-electrodes arranged in an 8×8 grid in the center of the probe. This array of electrodes constitutes the active region of the device, suitable for cell plating. A glass ring (6 mm high) mounted over the glass plate allowed the containment of the cell-bathing medium. The inter-electrode distance was 200 µm, the electrode diameter was 30 µm. Electrodes, contact pads and tracks were made of Titanium Nitride (TiN), with an internal reference electrode for grounding the bath. The impedance of each electrode was 30-50 kΩ. The MEA socket in the base plate featured a resistive heating element and a Pt100 temperature sensor.

Supplementary Figures

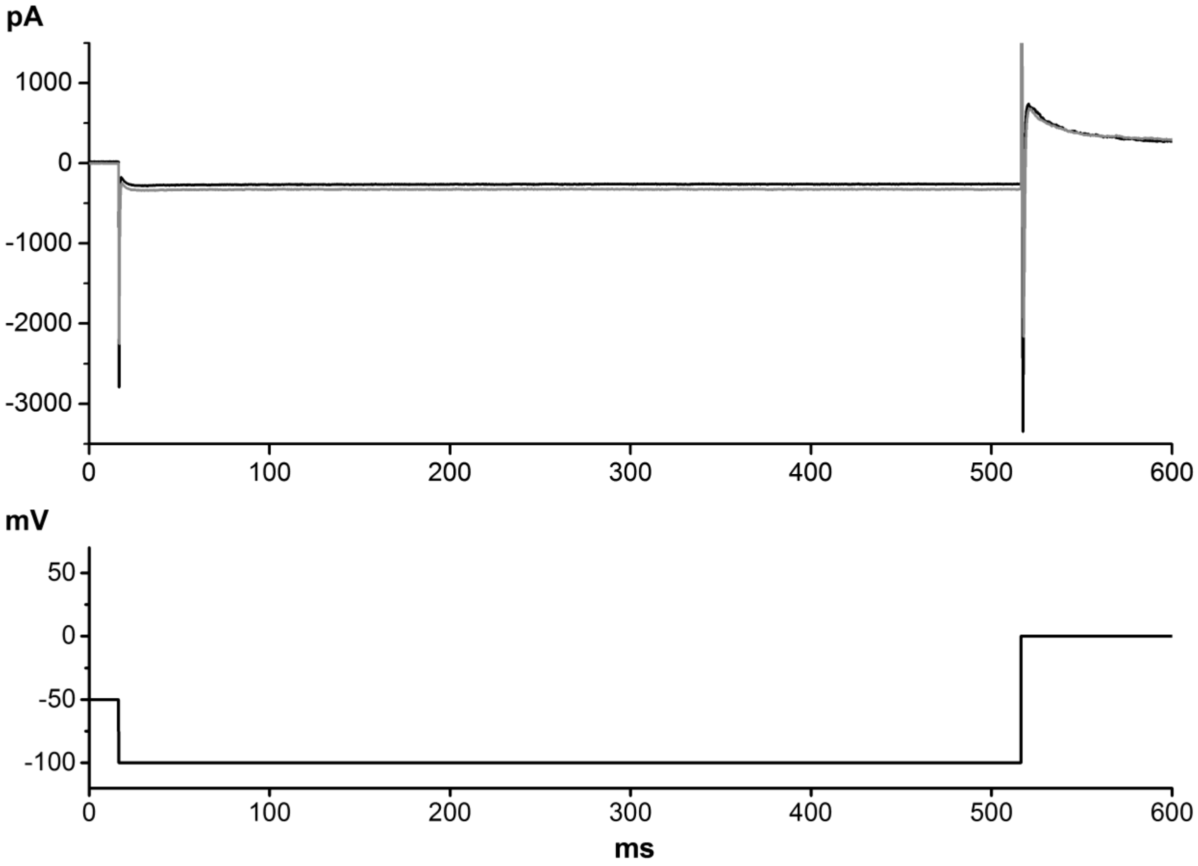

Fig. SM 1. Voltage clamp protocols applied to the cell of Fig. 1B in order to test membrane properties before (black) and after (gray) stimulation with the NPs. Lower: voltage clamp protocol. Upper: membrane currents. The fast inward transients correspond to the activation of a voltage-dependent inward current.

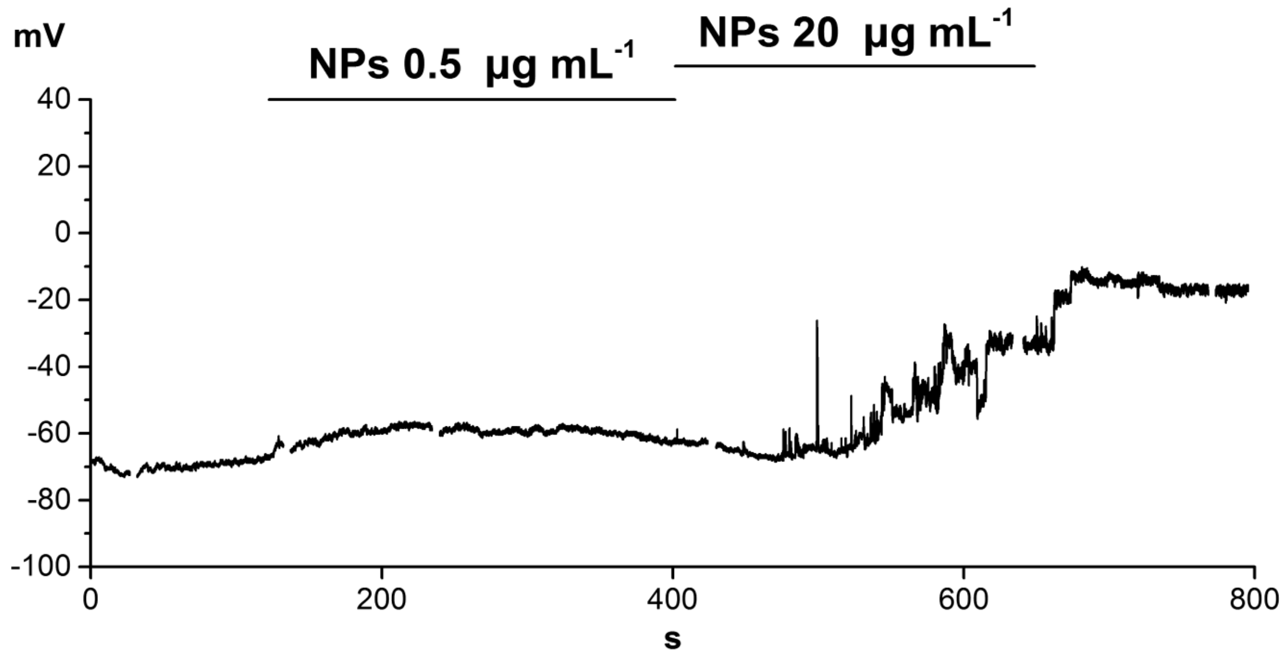

Fig. SM 2. Stimulation of a GT1-7 cell with NPs at the concentration of 0.5 μg mL^{-1} induced a small and reversible depolarization. Subsequent application of 20 μg mL^{-1} of the same NPs elicited a stronger response.

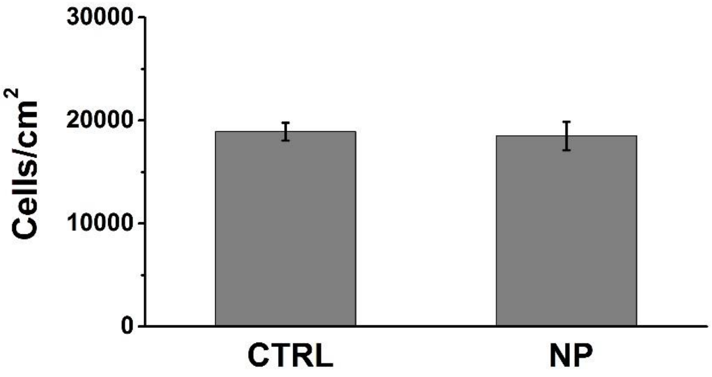

Fig. SM 3. GT1-7 cell survival after 24 hrs in 0% FBS was not affected by incubation with 20 µg mL^{-1} NPs (*t*-test, $p = 0.806$ two tailed; unpaired data, $n_{ctrl} = 6$, $n_{np} = 9$; normality of data has been previously assessed through a Shapiro-Wilk test).
